# Diagnosing chronic endometritis: when simplification fails to clarify

**DOI:** 10.1093/hropen/hoac023

**Published:** 2022-06-07

**Authors:** Maximilian Murtinger, Barbara Wirleitner, Dietmar Spitzer, Helena Bralo, Susanna Miglar, Maximilian Schuff

**Affiliations:** Next Fertility IVF Prof. Zech—Bregenz, Bregenz, Austria; Next Fertility IVF Prof. Zech—Bregenz, Bregenz, Austria; Next Fertility IVF Prof. Zech—Salzburg, Salzburg, Austria; Next Fertility IVF Prof. Zech—Bregenz, Bregenz, Austria; Next Fertility IVF Prof. Zech—Salzburg, Salzburg, Austria; Next Fertility IVF Prof. Zech—Bregenz, Bregenz, Austria

**Keywords:** chronic endometritis, plasma cell, CD138^+^, overdiagnosis, reproductive immunology

## Abstract

Reproductive immunology has grown in importance in recent years and has even developed into a discipline of its own within the field of reproductive medicine. Many aspects of reproductive failure such as repeated implantation failure or recurrent miscarriages are, meanwhile, seen as a consequence of aberrant expression of immunological factors. This is reflected by the increasing number of tests for assessing and quantifying different immune cell types as well as by a wide range of immune therapies offered to a clientele consisting of desperate patients requesting additional ‘IVF tools’: first, what is still usually disregarded is the enormous plasticity and fluctuation of most immune cells in the genital tract; second, their still poorly characterized functions in the endometrial cycle: further, their partially unknown role in embryo implantation and in establishing a pregnancy; and third, the fact that one of the fundamental hypotheses of reproductive immunology—of note—the Medawar concept or ‘Medawar's Paradox’ of semi-allogeneic graft embryo, is partially based on an erroneous assumption, i.e. the immunologic rejection and tolerance of an embryo. In the present opinion article, we comment on the diagnostic procedures and therapy approaches for chronic endometritis within the scope of reproductive medicine.

## Introduction

Many aspects of reproductive immunology principles have been applied to the clinical management of recurrent pregnancy loss, recurrent implantation failure (RIF) and IVF cycles that have failed for other reasons. The most illustrative example of this is the diagnosis and management of chronic endometritis (CE). Using this term, a total of 70 matches can be found in the 2021 PubMed database, whereas only 11 can be found in 2001. This indicates that CE diagnostics is gaining more and more importance. Even more attention is paid to this assumed aspect of sub- or infertility in the German-speaking countries of Austria, Germany and Switzerland. In these countries, the diagnosis and therapy of CE has already been included in the current guideline on recurrent miscarriage (RM) (Hennessy *et al.*, 2021). In case of RM, endometrial biopsy can be performed to exclude CE by immunohistology for CD138 (syndecan-1), a type I transmembrane heparan sulfate proteoglycan and a hallmark of plasma cells (PC). In addition, antibiotic regimens may be performed in case of RM and CE to prevent miscarriages (Toth *et al*., 2018). However, the current data are still inconclusive, and, to date, there are no uniform diagnostic criteria for CE. Below, we summarize all the current concepts of diagnosing CE and all the other aspects that should be considered before diagnosing and treating.

## The long road toward definition and therapy of CE

By definition, endometritis is an inflammation or irritation of the lining of the uterus. Normally, the underlying causes of endometritis—a bacterial or, sometimes, viral infection—are mostly prevented by endometrial shedding during menstruation and the ‘spatial’ subdivision of the female reproductive tract, consisting of two regions with different immune cell constitutions, the upper area (endocervix, uterus, and oviduct) with low-mass microbiome, and the high-mass-microbiome-enriched lower area namely vagina and ectocervix (Benner *et al.*, 2018; Barrios De Tomasi *et al.*, 2019). The cervical epithelial cells provide both a physical barrier made up of mucus and epithelial sheets and an immunological barrier consisting of cells with immune regulatory functions (Barrios De Tomasi *et al.*, 2019). In theory, inflammation can only occur when these natural barriers are impaired. This applies primarily to events related to an abortion or birth. Postpartum or puerperal endometritis is a particularly severe condition and can quickly progress to toxic shock, necrotizing fasciitis and may have potentially life-threatening consequences. Prior to the adoption of aseptic techniques in hospitals, puerperal fever was one of the major causes of maternal death following childbirth in the 19th century. This situation was changed by the postulations of the Hungarian physician Ignaz Semmelweis on the etiology, concept, and prophylaxis of childbed fever (Semmelweis, 1861). For example, between 1840 and 1846, the 1st Maternity Division of Vienna's largest hospital (later place of work of Ignaz Semmelweis) had an average maternal mortality rate of about 10%. Almost all the deaths were due to puerperal fever (Loudon, 2013).

In contrast to this acute form of endometritis, its chronic form is mostly asymptomatic and has remained largely undefined during many decades of the 19th century. In 1911, the British obstetric physicians and gynecologists Archibald Donald and Fletcher Shaw stated: ‘In the whole domain of gynecology there are no cases so common as those which generally go by the name of “chronic endometritis”. This term has been commonly used to denote a class of cases which are clinically well known but difficult to define. That the whole subject area is still in a state of confusion is apparent to everyone whose duty it is to try and give a clear account of minor gynecology to medical students’ (Donald and Shaw, 1911).

This situation however changed with the pioneering work of the Bohemian gynecologist Fritz Hitschmann and the Austrian gynecologist Ludwig Adler. They completely revised the diagnostic term of CE including its numerous subclassifications and rejected the term *endometritis glandularis*, which has been coined earlier by the pathologist Carl Ruge, a cousin of the famous pathologist Rudolf Virchow (Ruge, 1880). Hitschmann and Adler realized for the first time that the underlying principles of certain specific histological observations are not of a pathological but of a physiological nature. Moreover, they were the first to recognize the important role played by PCs in endometrial inflammatory processes and postulated the presence of PCs in the endometrium as a unique criterion for diagnosing a patient with CE. ‘We are inclined to make the detection of plasma cells a diagnostic criterion for CE’ (Hitschmann and Adler, 1907).

**Figure 1.**
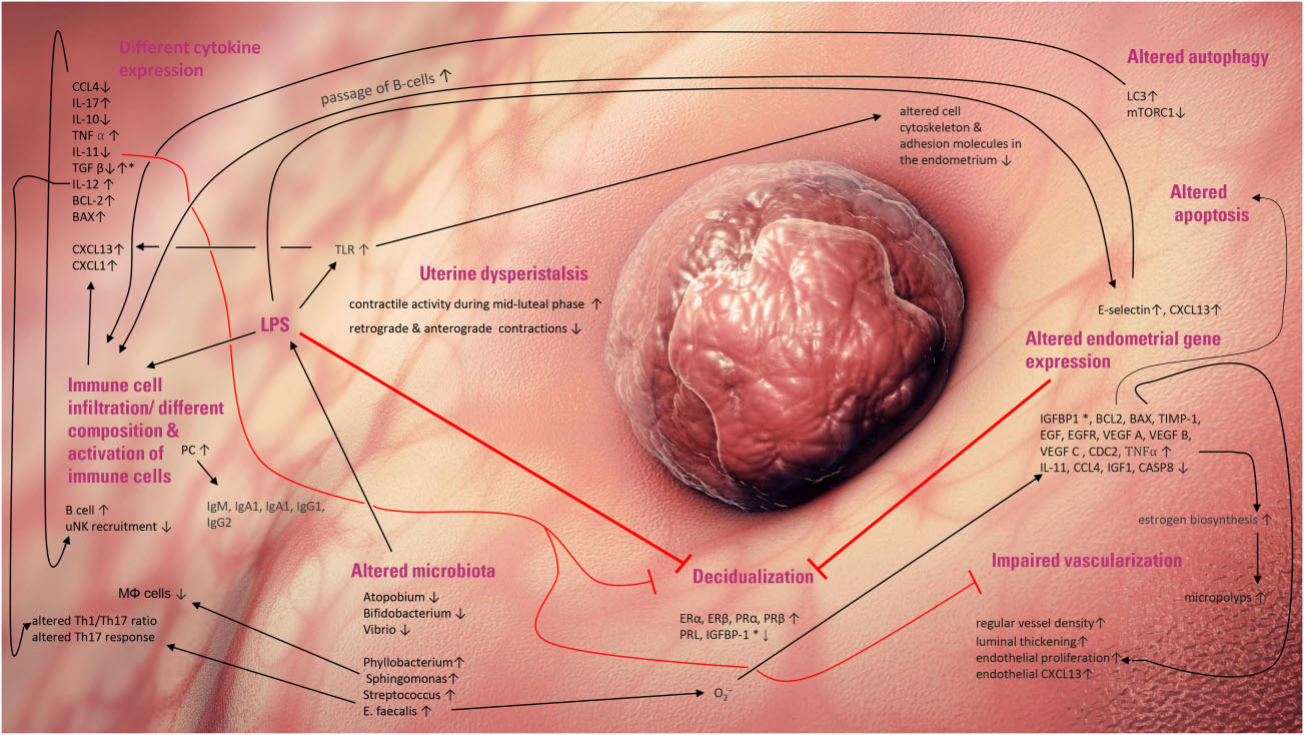
**Simplified illustration of postulated mechanisms how chronic endometritis (CE) might impact female fertility (modified from** **Buzzaccarini et al., 2020).** Reported pathophysiological effects of CE on immune cells and gene expression. contradictory results (marked by asterisks) were reported for IGFBP-1 (up- and downregulated and BAX (unaffected, respectively upregulated). BAX, Bcl-2-associated X protein; BCL2, B-cell lymphoma 2; CASP8, Caspase-8; CCL, Chemokine (C-C motif) ligands; CXCL, chemokine (C-X-C motif) ligand; EGFR, epidermal growth factor receptor; IGF-1, insulin-like growth factor 1; IGFBP-1, insulin-like growth factor-binding protein 1; IL, interleukin; MΦ, macrophages; LC3, microtubule-associated proteins 1A/1B light chain 3B; LPS, lipopolysaccharides; mTORC1, mTOR Complex 1; $O_{2}^{-}$, superoxide; PC, plasma cells; TIMP-1, TIMP metallopeptidase inhibitor 1; TGF β, transforming growth factor beta; uNK, uterine natural killer cells; Th, T helper cells; TLR, Toll-like receptors.

Until today, this remained the sole valid diagnostic criterion for CE. This is also due to the lack of symptoms in CE. While CE might cause abnormal uterine bleeding or unclear pelvic discomfort, it is asymptomatic in most cases. At the beginning of the new millennium, endometrial aspects of infertility gained more interest—a fact reflected by the wide range of endometrial receptivity tests offered. After having been neglected for many decades, CE diagnosis, too, shifted more and more into the center of interest. However, this poses several problems. There are many theories postulating that CE might negatively affect female fertility and the course of pregnancy. These theories encompass an altered endometrial decidualization (Wu *et al.*, 2017); changes in the endometrial gene expression profile *(*i.e. insulin-like growth factor-binding protein 1, B-cell lymphoma 2 (BCL-2), Bcl-2-associated X, insulin-like growth factor 1) (Di Pietro *et al.*, 2013); different composition of immune or immunomodulating cells such as B cells; natural killer cells (NK cells); regulatory T cell (Treg); or T helper cell subpopulations Th1/Th2, Th17 (Buzzaccarini *et al.*, 2020; Chen *et al.*, 2021; Kitazawa *et al.*, 2021), cytokine dysregulation, an altered autophagy (Wang *et al.*, 2019) and different microbiota of the female reproductive tract (Tanaka *et al.*, 2022); impaired vascularization or uterine dysperistalsis (Mount *et al.*, 2001). It should be noted that all these different issues might affect female fertility in multiple ways, cannot be seen separately and might interfere with each other. For example, lipopolysaccharides (LPS) of microorganisms might regulate cytokine expression, which in turn regulate leukocyte infiltration. An LPS-dependent Toll-like receptor activation might also reduce embryo attachment due to altered expression of adhesion molecules in human endometrial cells (see Fig. 1). All these issues of CE and infertility, of note implantation failure, are summarized in detail in the recent review of Buzzaccarini *et al.* (2020). However, robust studies that would substantiate these claims are still lacking—not least due to the fact that endometrial transcriptome analysis and immune cell diagnostics are far from being simple and calls for caution are needed in interpreting the results.

In addition, even the histological-based diagnosis of CE has its pitfalls. The diagnosis of CE is biased by several parameters, e.g. high inter-observer and intra-observer variabilities, the experience of the pathologist, or different staining methods (Mount *et al.*, 2001; Margulies *et al.*, 2021). Immunostaining for the canonical plasma cell marker CD138 is highly recommended to guarantee a reliable detection of PCs. But still, abortive residues and cervical contaminations could falsify the results as both cervical PCs and placental (trophoblast) residues express syndecan-1 (Groth, 2018). The most severe limitation of CE diagnosis, however, is the lack of consensus regarding the number of PCs needed for correct diagnostics (Mount *et al.*, 2001). A recent review and meta-analysis addressed this issue in detail (Huang *et al.*, 2020). The authors pilloried the huge variation of diagnostic criteria. For the 12 studies included in this meta-analysis (one case–control study, five retrospective studies and six prospective studies), six different diagnostic criteria for CE were applied. Therefore, the authors called for a consensus on the diagnostic criterion for CE (Huang *et al.*, 2020).

Based on only a small amount of data, the prevalence of CE differs tremendously, not only between the different subpopulations but also within the same subpopulations of infertile patients (Table I). Surprisingly, there is no robust study analyzing the potential occurrence of endometrial PCs in fertile women with a special focus on the different phases of the menstrual cycle. Though often neglected, there are some indications that PCs could appear in the endometrium of fertile women (Achilles *et al.*, 2005). Regardless of the lack of robust data, it is difficult to understand that not a few pathologists use the presence of a single endometrial PC for making the diagnosis (Margulies *et al.*, 2021). A recently published prospective, multi-center study encompassing a cohort of 80 young infertile patients revealed that in more than half of the study participants, ≥1 PC could be found. Moreover, the number of PCs present failed to predict live birth (Herlihy *et al.*, 2022). Although other studies reached opposite conclusions, the fact remains that the overall data regarding this issue are still sparse (McQueen *et al.*, 2015; Li *et al.*, 2021). Due to the lack of standardized histological criteria, hysteroscopy has increasingly been proposed as an important aid for CE diagnosis. Thereby, diagnostic hysteroscopy has demonstrated high diagnostic accuracy for several uterine and endometrial pathologies including benign conditions such as myomas, endometrial polyps or endometrial hyperplasia but also endometrial cancer (Gkrozou *et al.*, 2015). Hysteroscopy, performed within the proliferative phase of the menstrual cycle, might also allow the identification of signs of endometrial inflammation. Classical hysteroscopic finding of CE often includes an endometrial ‘strawberry pattern’ with large areas of hyperemic endometrium flushed with white central points. However, from our clinical experience and according to the literature, one might assume that this observation is in fact a rather rare finding.

**Table I:** Prevalence rates of chronic endometritis according to the literature.

Population	Prevalence	References
Infertile women	0.2–56.8%	Wild (1986), Sahmay *et al.* (1995), Féghali *et al.* (2003), Polisseni *et al.* (2003), Cicinelli *et al.* (2005), Chen *et al.* (2016) and Cicinelli *et al.* (2018)
Recurrent implantation failure	14–67.5%	Johnston-MacAnanny *et al.* (2010), Cicinelli *et al.* (2015), Bouet *et al.* (2016), Kitaya *et al.* (2017), and Zhang *et al.* (2019)
Recurrent miscarriage	9.3–67.6%	Kitaya (2011), Zolghadri *et al.* (2011), Cicinelli *et al.* (2014), McQueen *et al.* (2014), and Bouet *et al.* (2016)
Abnormal uterine bleeding	1.4–52%	Kitaya *et al.* (2018) and Song *et al.* (2018)
Patients with endometrial polyps	28–92.6%	Cicinelli *et al.* (2019), Kuroda *et al.* (2020), and Guo *et al.* (2021)

Sometimes CE was found to be associated with small mucosal proliferations (<1 mm), termed as micropolyps, stromal edema (pale and thickened) and endometrial polyps in combination or alone. The latter endometrial polyps however seem to have low diagnostic accuracy for CE (Tsonis *et al.*, 2021). Although no consensus exists in regard to hysteroscopy-based CE criteria and differences reported in sensitivity and specificity of the afore mentioned criteria, one might assume that CE is not present when at least one of the three other hysteroscopic CE criteria could not be confirmed (Cicinelli *et al.*, 2005; Tsonis *et al.*, 2021). Although discussed with some controversy, hysteroscopy performed by experienced staff, combined with histological evaluation should be considered as a diagnostic tool for the diagnosis of CE (Puente *et al.*, 2020; La Marca *et al.*, 2021).

Seeing these pitfalls of the accuracy in CE diagnostics, the current data are even more limited when it comes to the treatment of CE. Therefore, the serious lack of evidence in terms of the occurrence of endometrial pathogens as a trigger of infection is a major problem here. In principle, the CE diagnosis needs first the verification of ascending pathogens. In fact, this is often not done since it is considered a rather complicated procedure, involving sampling the potential pathogens from the uterus with low-mass microbiota through high-mass microbiota of the vagina—bearing the danger of contamination. Moreover, the verification of pathogens failed since certain bacterial strains are hard to cultivate. The mere detection of certain 16S rRNA sequences in the mucous membrane does not differentiate between living bacteria or (dead) bacterial fragments. This may, thus, lead to misdiagnosis—although it cannot be excluded that even inactive bacterial fragments can still contribute to a physiologic interaction with host cells (Benner *et al.*, 2018), and thereby influencing female fertility. In the clinical practice of CE management, this often results in the blind application of broad-spectrum antibiotics without the patients being tested positive for the presence of the relevant pathogens. From a medical point of view, this undermines the principles of good clinical practice, diagnostics and subsequent therapy. The diagnosis will be proven by the cure rates (in Latin: *diagnosis ex-juvantibus*—gaining diagnosis by successful therapy). The endpoint of successful therapy is not anymore the eradication of the (still unproven) pathogen but the absence of PCs. This approach might raise doubts, especially regarding the possible side effects from altered intestinal and vaginal flora and the potential candida infections associated to them. Moreover, the same inconsistency as observed in CE diagnostics can also be found in therapy regimens with different types of antibiotics, different dosages, and therapy durations. Even the routes of applications (including intrauterine flushing) were found to be inconsistent (Sfakianoudis *et al.*, 2018; Huang *et al.*, 2020). One point worthy of note is that the cure rates were reported to range from 59% to 99%. But, no robust randomized trials have been conducted to demonstrate any advantage resulting from the administration of antibiotics. The fact that even the application of broad-spectrum antibiotics such as Doxycycline or Amoxicillin for 12–14 days often does not erase the endometrial PCs undermines the theory of bacteria-induced CE. It should be acknowledged that inflammation might also have non-microbial causes. Inflammation without apparent pathogenic infection, often designated as ‘sterile inflammation’, has not only been implicated in pathological conditions such as cardiovascular diseases, pulmonary disorders and cancer but is suggested to be involved in (patho)physiological conditions of reproduction such as preeclampsia, preterm labor, intrauterine leiomyomas, endometrioses and embryo implantation (Negishi *et al.*, 2021).

However, these aspects are usually not taken into consideration in CE therapy. Instead of questioning current CE dogmas, modified antibiotic combination regimes are often used therefore to treat cases of PC persistence (i.e. Metronidazole and Ciprofloxacin or Minocycline and Doxycycline combined). Adding to this is the fact that there is a lot of hype (with rising tendency) around the importance of the uterine microbiome for reproductive health. Regardless of the question whether this is justified or not, we should honestly ask ourselves if we do more harm than good by pursuing such therapy approaches.

## *In vitro* fertilization—different aspects of overdiagnosis

There is, without doubt, robust evidence that too many patients are being overdiagnosed. In principle, this fact is due to two major causes: overdetection and overdefinition of a disease. Although it is still difficult to arrive at a satisfactory definition of the term overdiagnosis and to draw sharp boundaries between diagnosis and overdiagnosis, there is no doubt that overdiagnosis is a serious problem in healthcare. It can harm patients by diagnosis-related anxiety, the overmedication or diagnostic or therapy-related depression (Kale and Korenstein, 2018). Overmedicalization represents a severe financial burden for the healthcare systems, too. In the USA alone, an estimated amount of up to $46 billion are wasted on unnecessary treatments every year (Rothberg *et al.*, 2014).

Overdiagnosis might result from (i) increasingly sensitive tests, (ii) incidental findings or (iii) excessively widened definitions of diagnostic criteria (Moynihan *et al.*, 2012). Prominent examples are bone mineral density screening for osteoporosis in younger patients with no risk factors or cholesterol screening among asymptomatic patients. Meanwhile, the problem of overdiagnosis has also reached the field of reproductive medicine. The broad application of preimplantation genetic testing for aneuploidies (PGT-A) with high-resolution next-generation sequencing (NGS) platforms perfectly meets the above-mentioned criteria. PGT-A using NGS technology disproved the dogma that human embryos are either uniform euploid or aneuploid. Instead, it has been demonstrated that a not insignificant portion of human embryos can be classified as chromosomal mosaics (CM), having a mixture of both abnormal and normal cells. The given thresholds for euploid (i.e. <20% aneuploid cells), aneuploid and mosaic embryos were set without biological but technical criteria (determined by the number of trophectoderm cells—normally five cells—biopsied). This resulted either in the discarding of thousands of normal embryos with normal pregnancy potential or in the transfer of CM embryos, leaving patients exposed to high psychological stress after the transfer (Murtinger *et al.*, 2018; Gleicher *et al.*, 2018). Preimplantation testing is, however, a prominent but not the only example for overdiagnosis in reproductive medicine.

The above-listed items (increasingly sensitive tests, incidental findings or excessively widened definitions of diagnostic criteria) also apply to the current diagnostic criteria for CE. First, the CD138 immunohistochemistry increases the sensitivity of finding PC. Second, if you look for something, you will find something. Diagnostic screening may reveal ‘incidental findings’ in individuals being tested for other reasons. It is well accepted that RIF and RM are frustrating and count among the most difficult issues in reproductive medicine because their etiology often remains unknown. The detection of endometrial PCs might represent such an incidental finding. The presence of PCs might not be necessarily associated with RIF or RM. Third, the excessively widened definitions of diagnostic criteria might also be applicable for CE diagnosis when a single or just a few PCs are suggested to provide sufficient evidence for CE diagnosis.

## Reproductive immunology revisited

Apart from chromosomal instability during early embryogenesis, certain uterine factors (myoma, fibroids, polyps) and inadequate endometrial-embryonic synchronization processes, certain immunological aspects are also suggested as being responsible for embryo loss in human reproduction. The reasons for this are obvious. Infiltrating immune cells represent a major cellular component of the maternal decidua. Furthermore, studies have shown that specific immune cells in rodents are indispensable for achieving and maintaining a pregnancy. It is, therefore, beyond doubt that a functional responsive immune system is crucial for the establishment of a successful pregnancy.

Reproductive immunology already attracted closer attention in the 1950s, provoked by the definition of the immunological paradox of pregnancy and the postulation of the semi-allograft concept by the famous Brazilian-British biologist Sir Peter Medawar. Medawar’s life’s work still represents the basis for many aspects of modern immunology and transplantation medicine. However, regarding the semi-allograft concept, Medawar was probably wrong. The suggestion that implantation failure, miscarriage and preterm birth occur as a result of maternal immunosuppression failure, leading to the rejection of the embryo, was taken up with enthusiasm. Parallels between transplantation immunology and reproductive immunology were drawn—probably based on wrong assumptions. As a fatal consequence, the field of transplantation medicine still serves as a blueprint for many reproductive aspects that cannot be investigated *in vivo* such as the process of embryo implantation, adhesion and invasion as well as implantation failure and miscarriage. Still, immune-modulating therapies such as immunoglobulins, intralipid infusion, application of granulocyte colony-stimulating factor, peripheral blood mononuclear cells, subcutaneous administration of TNF-alpha inhibitors, leukemia inhibitory factor, oral administration of anti-inflammatory-acting glucocorticoids and even immunosuppressant drugs used for transplanted patients (such as tacrolimus—a macrolide lactone) (Nakagawa *et al.*, 2015) are offered to RIF and RM patients on a more or less regular basis. According to recent studies, evidence of their effectiveness is lacking, and they are not recommended by the authors (Mascarenhas *et al.*, 2021). In this context, it is equally important to take into account recent findings that, from an evolutionary view, embryo implantation might derive from an ancestral inflammatory process. Therefore, a pro-inflammatory process is the first step and plays a pivotal role in mammal pregnancy (Chavan *et al.*, 2017). This, in consequence, does not only entail the risk of therapeutic ineffectiveness due to a wrong theory but may also increase the risk of jeopardizing the patients’ health and the desired IVF outcome.

Likewise, many diagnostic immunological tests are meanwhile offered to IVF patients—not only the testing of PCs but also of other lymphoid cells like Tregs, T cells or uterine natural killer cells (uNK). Particularly the latter have evoked a lot of interest, as NK cells constitute 50–90% of the leukocytes in the decidua and the fact that NKs have been suggested to be mediators of cellular cytotoxicity. Therefore, an elevated uNK level is generally regarded as having a detrimental impact on establishing and maintaining a pregnancy. However, this raises several questions. First, the variation in the number of uNKs within different reproductive phases is stunning. Their number increases dramatically from proliferative to the late secretory phase of menstrual cycle. In addition, the uNK cell development is highly dynamic during gestation showing phenotypic differences reflected by alternations in gene expression. For example, killer cell immunoglobulin-like receptors (KIR) expression is reduced from the 6th to 12th weeks of pregnancy, while NKG2D and NKp80 expression increases in the second trimester of pregnancy (Bulmer and Lash, 2019).

However, the most critical issue is the fact that the NK cell lineage is comprised of a relatively heterogenous and diverse population of CD56^+^/CD3^−^ cells (Cooper *et al.*, 2001). The endometrium almost exclusively contains CD56^bright^CD16^−^ NK cells. This NK population however does not only show higher CD56 expression compared to peripheral NK cells (CD56^dim^) but expression of CD9, CD49a and the immunosuppressive molecule PP14. Uterine NKs also demonstrate significant differences in expression pattern compared to peripheral NK cells. Koopman and colleagues found at least 278 genes with ≥ threefold change in their expression compared to peripheral NKs (Koopman *et al.*, 2003). Furthermore, although they can acquire cytotoxic ability when decidua is infected, it is assumed that may have a rather immunomodulatory role instead of cytotoxic effector responses (summarized in Gaynor and Colucci, 2017)—findings that have already been made by the beginning of this millennium—but are often neglected. Meanwhile, research provides a clearer picture how uNKs are assumed to contribute to fundamental physiological processes of pregnancy within the decidua. Uterine NKs are involved in plenty of physiological processes in establishing and maintaining pregnancy. They do not only trigger the invasion of the extravillous trophoblast (EVT) through direct interaction with the fetal trophoblast cells but also regulate the depth of invasion by balancing between enhancing and inhibiting EVT invasion (Gaynor and Colucci, 2017). They also secrete matrix metalloproteinases—thereby contributing directly to decidua-associated vascular remodeling—and several angiogenic factors including vascular endothelial growth factor, placental growth factor and angiopoietin 1/2, and indirectly modify spiral arteries through their interaction with EVT (Gaynor and Colucci, 2017). In turn, trophoblast major histocompatibility complex class I antigens may modulate the uNK cell activity. This also holds true for the interaction of different immune cells. For example, NK cells are important in the regulation of TH17 cells; while in turn, Th17 cells induce the activation of uNK cells. Focusing on only one immune cell population in such complex interacting pathways bears the danger of misrepresenting their true nature.

At least, it can be assumed with adequate probability that uNKs do not represent a uniform and cell population. Not only the dynamics of gene expression during gestation stresses the picture that uNK represents a uniform NK class. A recent single-cell RNA sequencing-based study indicates the existence of different sub-populations of uNK cells (Vento-Tormo *et al.*, 2018).

Although evidence for their lymphocyte origin was found in the 1960/70s and our understanding of development and function of NK cells has progressed significantly in recent years, we must face the fact that we are just beginning to understand their enormous plasticity and their diverse functions. It is even still unclear whether locally secreted chemokines/cytokines attract NK cells to the endometrium, where they undergo a local differentiation or if uNKs arise from progenitors in the endometrium. The problem of investigating uNKs *in vivo* hampers uNK research. While data from animal model systems helped to resolve many open issues; meanwhile, it is clear that fundamental differences between species exist. For example, when comparing mice and humans: in both species, uNKs contribute to fundamental physiological processes of pregnancy within the decidua, but there are obvious key differences in how these effects are mediated (Gaynor and Colucci, 2017).

Does it make sense to quantify such a highly dynamic cell population with high plasticity that is still of unknown origin and development with mostly unknown functions*?*

Applied to the situation of CE and endometrial PCs, it should not be assumed that the complexity of (endometrial) PCs is less complex compared to (uterine) NK cells. PCs are differentiated antigen-activated B lymphocytes. They can secrete large amounts of—different—antibodies and are, thus, an integral and effective part of humoral immunity. However, it is now recognized that they are also important cytokine producers being involved in physiological processes—independent of antibody secretion such as regulation of hematopoiesis, gut homeostasis, and others (Pioli, 2019). This also holds true for certain oncological processes. PCs can also be found in solid tumors where they negatively affect anti-tumor, T-cell-mediated immunity (Shalapour *et al.*, 2015). However, it must be acknowledged that probably most antibody-independent functions remain to be elucidated. Furthermore, the different roles of PCs might also be reflected by a still inconceivable heterogeneity in PC subpopulations (Delaloy *et al.*, 2022).

## Endometrial PCs—the open issues

The question remains open as to whether the presence of endometrial PCs represents a pathological situation. Although large-scale studies are still lacking, there are hints that endometrial PCs can also be found in physiological situations (Achilles *et al.*, 2005). It should also be remembered that many mucosal layers encompass mainly IgA-producing PCs. This includes the lamina propria along the gastrointestinal tract and lacrimal, nasal, and salivary glands in the upper airways. Interestingly IgA antibodies are suggested not to be restricted to inflammatory functions but might have anti-inflammatory properties too (Monteiro, 2014). Thus, there are some indications that IgA releasing PCs do not only keep pathogens at bay but protect the commensal microbiota (Bemark and Angeletti, 2021). It may appear highly speculative to assume that a low PC count in the endometrium can be protective for the microbiota of the uterus. However, we urgently need to grasp the importance of achieving a broader understanding in reproductive immunology.

## Conclusions

More than 115 years after the implementation of the Hitschmann–Adler criteria for CE, a revision of CE definition is urgently needed. Future CE criteria must not be based on a sole criterion but should include the hysteroscopic findings. In a much broader context, add-on immunological tests and treatments should be omitted. Immune- ‘add-ons’ should not be offered to patients, not even within the scope of a trial—at least not until we are able to understand the basics of reproductive immunology and rule out the risk of harming patients in the process. It is time to banish this simplified way of thinking from our minds. While it may be reasonable that the complexity of reproductive immunology is broken down to findings such as elevated uNK cell counts, Th1/Th2 ratios and presence of PCs, this does not reflect human biology and does not satisfy the requirements of modern reproductive medicine.

## Data availability

No new data have been generated or analyzed in support of this publication.

## Authors’ roles

M.M., B.W. and M.S. contributed to design, data collection and interpretation of current evidence, along with drafting the manuscript. D.S., H.B. and S.M. contributed substantially by critically revising the manuscript and adding important aspects.

## Funding

None.

## Conflict of interest

The authors declare no competing interests.
